# Lack of Competence of US Mosquito Species for Circulating Oropouche Virus

**DOI:** 10.3201/eid3103.241886

**Published:** 2025-03

**Authors:** Anne F. Payne, Jessica Stout, Peter Dumoulin, Timothy Locksmith, Lea A. Heberlein, Molly Mitchell, Arnold Rodriguez-Hilario, Alan P. Dupuis, Alexander T. Ciota

**Affiliations:** Wadsworth Center, New York State Department of Health, Slingerlands, New York, USA (A.F. Payne, J. Stout, A.P. Dupuis II, A.T. Ciota); Florida Department of Health, Tampa, Florida, USA (P. Dumoulin, T. Locksmith, L.A. Heberlein, A. Rodriguez-Hilario); Florida Department of Health, Jacksonville, Florida, USA (M. Mitchell); University at Albany School of Public Health, Rensselaer, New York, USA (A.T. Ciota)

**Keywords:** Oropouche virus, viruses, vector-borne infections, vector competence, virus emergence, United States

## Abstract

Given recent outbreaks of Oropouche virus in Latin America and >100 confirmed travel-associated cases in the United States, we evaluated the competence of US vectors, including *Aedes albopictus*, *Culex quinquefasciatus*, *Culex pipiens*, and *Anopheles quadrimaculatus* mosquitoes. Results with historic and recent isolates suggest transmission potential for those species is low.

Oropouche virus (OROV) is a negative-sense, segmented RNA virus and a member of the family Peribunaviridae, genus *Orthobunyavirus*. OROV was first identified in Trinidad and Tobago in 1955, and although it was previously detected in several countries in Latin America, large outbreaks have historically been limited to the Amazon region of Brazil (*1*,*2*). In 2024, >10,000 cases were reported, with unprecedented activity outside Brazil (*3*). In addition, 108 imported cases have been identified in travelers or residents returning to the United States. Although most of those cases have been in Florida, cases have also been identified in New York, New Jersey, Kentucky, Colorado, and California (*3*). 

OROV infection is generally associated with a mild, self-limiting febrile illness, yet more extensive disease, including fatal infections, has been attributed to the recent outbreak. Furthermore, vertical transmission and associations with congenital abnormalities and fetal death have been reported (*4*,*5*).

Although the primary vector of OROV is *Culicoides paraensis* midges (*6*), mosquitoes have also been implicated, particularly *Culex quinquefasciatus* mosquitoes (*7*). However, experimental assessment of OROV transmission by mosquitoes is limited (*8*–*10*). We investigated whether prominent US mosquito species have the potential to contribute to local maintenance. 

We isolated OROV from reverse transcription PCR–positive serum from a febrile patient from Cuba after amplification on Vero cell culture (OROV 240023). We obtained RNA through the QIAGEN QIAcube using the DSP Viral RNA Mini Kit (https://www.qiagen.com). We completed first and second strand cDNA synthesis with random primers using NEB ProtoScript II First Strand cDNA Synthesis Kit and NEBNext Ultra II Non-Directional RNA Second Strand Synthesis Module (New England BioLabs, https://www.neb.com). We completed purification using AMPure bead-based methods (Beckman Coulter, https://www.beckmancoulter.com) and completed library preparation using the Illumina DNA Prep kit (Illumina, https://www.illumina.com). We completed sequencing on an Illumina NextSeq 1000 with fastq files generated through BaseSpace. We achieved reference-based assembly using an in-house pipeline. We aligned high-quality reads to reference sequences (GenBank accession nos. PQ064919.1, PQ064920.1, and PQ064921.1) and generated consensus sequences. We compared OROV 240023 to the 1955 strain OROV TRVL9760 (GenBank accession no. KC759122–24) after pairwise alignment in Geneious Prime (https://www.geneious.com). OROV 240023 was 6.3% divergent on the nucleotide level and 0% on the amino acid level in the small segment, 5.2% divergent on the nucleotide level and 1.8% on the amino acid level in the medium segment, and 10.9% divergent on the nucleotide level and 2.0% on the amino acid level in the large segment. The 69 unique amino acid residues are dispersed throughout the medium and large peptides. 

Using OROV 240023 and TRVL9760, we assessed competence of mosquitoes after feeding on blood meals containing 6.5 log_10_ PFU/mL with 4 US species: *Aedes albopictus*, *Cx. quinquefasciatus*, *Cx. pipiens*, and *Anopheles quadrimaculatus*. *Ae. albopictus* mosquitoes, F46, were collected in Suffolk County, New York , in 2014. *Cx. quinquefasciatus* mosquitoes, F57, were collected in Chattooga County, Georgia, in 2022. *Cx. pipiens* mosquitoes, F50, were collected in Albany County, New York, in 2022. *An. quadrimaculatus* mosquitoes (BEI Resources, https://www.beiresources.org) were originally collected in Orlando, Florida, in 1930. At 14 days postinfection, we anesthetized mosquitoes and assessed competence using 26–50 mosquitoes/population. We removed legs and placed the proboscises in a capillary tube containing 25% sucrose. After 30 minutes, we collected secretions and bodies and stored all samples −70°C. We thawed bodies and legs, homogenized for 30 seconds with a stainless-steel bead (Daisy, https://www.daisy.com) using a Retsch Mixer Mill (https://www.retsch.com), and centrifuged for 2 min at 10,000 × *g*. We tested samples by plaque assay on Vero cells to determine infectivity (positive body), dissemination (positive legs), and transmission potential (positive saliva).

Our results suggest a general lack of competence for all species with both viral strains (Table). Infection rates for *Ae. albopictus* mosquitoes were 2.0% for TRVL9760 and 0.0% for OROV 240023, for *An. quadrimaculatus* mosquitoes 4.0% for TRVL9760 and 0.0% for OROV 240023, for *Cx.* q*uinquefasciatus* mosquitoes 2.0% for TRVL9760 and 0.0% for OROV 240023, and for *Cx. pipiens* mosquitoes 0.0% for OROV TRVL9760 and 2.0% for OROV 240023. Although strain-dependent, those data represent a single positive mosquito for each species. Of the 3 TRVL9760-positive mosquitoes, 2 had disseminated infections, and no transmission was detected. Our results provide evidence of infectivity of OROV in *Anopheles* mosquitoes, and given the small sample sizes, modest transmission potential is possible for this species. A more comprehensive assessment of competence could be achieved with larger sample sizes. 

**Table:** Vector competence of US mosquito species for Oropouche virus

Strain	Input, log_10_ PFU/mL	Species	% Infected (no. tested)	% Disseminated (no. tested)	% Transmitted (no. tested)
OROV TRVL9760	6.5	*Aedes albopictus*	2 (50)	100 (1)	0 (1)
		*Anopheles quadrimaculatus*	4 (26)	100 (1)	0 (1)
		*Culex. quinquefasciatus*	2 (50)	0 (1)	0 (1)
		*Cx. pipiens*	0 (50)	0	0
OROV 240023	6.5	*Ae. albopictus*	0 (50)	0	0
		*An. quadrimaculatus*	0 (31)	0	0
		*Cx. quinquefasciatus*	0 (46)	0	0
		*Cx. pipiens*	2 (50)	100 (1)	100 (1)

OROV 240023 infection was only identified for *Cx. pipiens* mosquitoes, the only mosquito with positive saliva, indicating OROV transmission. McGregor et al. (*9*) demonstrated higher OROV TRVL9760 infection levels at the same dose in *Cx. quinquefasciatus* mosquitoes but similarly limited transmission potential. In addition, de Mendonça et al. (*10*) used a historic sloth isolate, OROV BeAn19991, and found a lack of infectivity in *Cx. quinquefasciatus* mosquitoes. Our data suggest that the currently circulating genotype remains limited in its capacity to infect that species. These results demonstrate some strain-specific variability in competence but suggest the likelihood of these species maintaining OROV in North America remains low.
